# Prevalence and Preoperative Characteristics Associated with Complicated Appendicitis Among Children Undergoing Appendectomy in Southwestern Saudi Arabia: A Hospital-Based Cross-Sectional Study

**DOI:** 10.3390/children13091275

**Published:** 2026-09-20

**Authors:** Salman M. Ghazwani, Mohammed A. Zailai, Othman Iskander, Jalal Abu Halimah, Ghazi I. Al Jowf, Salhah M. Ghazwani, Ahmed Mobarki, Dhiyaa A. H. Otayf, Khalid Mohammed Aldalgan, Majd Thunayyan Alhazmi, Sultan Mohammed Banser, Eyad Z. Omar, Saja A. Almraysi, Farjah H. Algahtani, Mohammad A. Jareebi

**Affiliations:** 1 Department of Surgery, College of Medicine, Jazan University, Jazan 82816, Saudi Arabia; sghazwani@jazanu.edu.sa (S.M.G.); doctorothman@hotmail.com (O.I.); jabuhalimah@jazanu.edu.sa (J.A.H.); 2 Faculty of Medicine, Jazan University, Jazan 45142, Saudi Arabia; dhiyaaot@gmail.com (D.A.H.O.); iiocraft629@gmail.com (K.M.A.); majdalhazmi1212@gmail.com (M.T.A.); eyadzaki42@gmail.com (E.Z.O.); sajaalasiri1@gmail.com (S.A.A.); 3 Department of Public Health, College of Applied Medical Sciences, University Medical Clinics Complex, King Faisal University, Al Hofuf 37912, Saudi Arabia; galjowf@kfu.edu.sa; 4 Paediatrics Department, King Fahd Central Hospital, Jazan Health Cluster, Jazan 45142, Saudi Arabia; salhaghazwany@gmail.com; 5 College of Pharmacy, Jazan University, Jazan 45142, Saudi Arabia; ahmedalmobarki@outlook.com; 6 Department of Pediatric Surgery, King Fahd Central Hospital, Jazan 45142, Saudi Arabia; sultan.ban50@gmail.com; 7 Oncology Center, Chair of Epidemiology and Public Health Research, Faculty of Medicine, King Saud University Medical City, King Saud University, Riyadh 12373, Saudi Arabia; falgahtani@ksu.edu.sa; 8 Department of Family and Community Medicine, College of Medicine, Jazan University, Jazan 82816, Saudi Arabia

**Keywords:** paediatric appendicitis, complicated appendicitis, appendiceal perforation, prevalence, peritonitis, associated factors, hospital-based cross-sectional study, Saudi Arabia

## Abstract

**Background/Objectives**: Complicated appendicitis carries greater morbidity than uncomplicated disease, yet its prevalence and determinants in southwestern Saudi Arabia are largely undocumented. This study estimated the prevalence of complicated appendicitis among children undergoing appendectomy and identified the preoperative characteristics independently associated with it. **Methods**: A hospital-based cross-sectional study included 275 children aged 14 years or younger who underwent appendectomy at King Fahd Central Hospital, Jazan, between January 2020 and December 2024. Complicated appendicitis was defined by appendiceal perforation, purulent intra-abdominal collection, or generalized peritonitis documented in the structured intraoperative record. Prevalence is reported with Wilson 95% confidence intervals. Adjusted prevalence ratios (aPR) with 95% confidence intervals were estimated by modified Poisson regression with robust variance, using preoperative variables selected for clinical plausibility. Two sensitivity analyses used broader outcome definitions. **Results**: Complicated appendicitis was present in 57 of 275 children, a prevalence of 20.7% (16.4–25.9). Prevalence did not differ by age group, sex, referral status, or symptom duration, but was higher among urban than rural residents (26.3% vs. 15.2%, *p* = 0.035). Two preoperative characteristics were independently associated with complicated disease: generalized abdominal tenderness (aPR 3.01, 1.75–5.20) and reported fever (aPR 1.87, 1.11–3.17). Symptom duration, clinical dehydration, leukocyte count, and admission haemoglobin were not independently associated with severity. Among children with imaging, appendiceal diameter contributed independently in an exploratory model (aPR 1.06 per mm, 1.02–1.11). Under broader outcome definitions, symptom duration and dehydration became significant while generalized abdominal tenderness remained associated throughout, indicating that several associations depend on how severity is defined. **Conclusions**: One in five children came to the operation with complicated appendicitis. Tenderness extending beyond the right iliac fossa, together with reported fever, was the most consistent bedside signal of advanced disease, whereas associations with delayed presentation, dehydration, and haemoglobin were sensitive to the outcome definition and require prospective confirmation.

## 1. Introduction

Acute appendicitis remains the most common condition requiring emergency abdominal surgery in children. Roughly 60,000 paediatric appendectomies are performed each year in the United States alone, and the lifetime risk approaches 7 to 8% [1]. Mortality has fallen to near zero in well-resourced systems, but the disease continues to consume substantial hospital resources, and the burden is concentrated in the subset of children whose appendix has already perforated by the time they reach an operating theatre [2].

Appendicitis is now understood as a heterogeneous condition rather than a single inevitable progression. Complicated disease, defined by perforation, intra-abdominal abscess, or generalized peritonitis, follows a distinct clinical course from uncomplicated inflammation [3]. Children with complicated appendicitis stay in hospital longer, require extended antibiotic therapy, and experience more postoperative infections than those operated on earlier in the disease process [4]. A nationwide Swedish cohort confirmed that this gradient in morbidity persists across an entire health system rather than reflecting the case mix of individual centres [5]. Identifying which children are likely to harbour advanced disease at the moment of presentation is therefore a practical priority, not an academic one.

Where that risk accumulates has been debated for two decades. Early work implicated in-hospital surgical delay as a driver of perforation [6], but subsequent studies found that short, structured waiting periods before appendectomy do not measurably increase severity when resuscitation and antibiotics are started promptly [7,8]. Attention has consequently shifted to the interval before hospital arrival. Prospective paediatric data show that perforation rates rise almost linearly with symptom duration, and that prehospital delay explains far more of the variation than in-hospital delay does [9]. Children presenting more than 24 h after symptom onset are substantially more likely to have complicated disease [10]. Alongside symptom duration, prediction efforts have focused on objective markers: appendiceal diameter and other sonographic features [11], inflammatory and nutritional biomarkers [12], and serum sodium, which performs well enough in meta-analysis to have entered several published scores [13].

Despite this literature, evidence from southwestern Saudi Arabia is limited. The Jazan region spans coastal plains, agricultural lowlands, and isolated mountain communities, many of which depend on a single tertiary referral hospital for paediatric surgical care. A recent Saudi series reported a higher proportion of complicated appendicitis than most international cohorts and identified younger age and higher Pediatric Appendicitis Scores as predictors [14], but the regional characteristics associated with severity have not been systematically examined, and no published work from this catchment has modelled admission symptoms, examination findings, and laboratory values together. This study therefore had two aims: to estimate the prevalence of complicated appendicitis among children undergoing appendectomy at a tertiary referral centre in Jazan, and to identify the preoperative clinical, laboratory, and imaging characteristics independently associated with it.

## 2. Materials and Methods

### 2.1. Study Design and Setting

This hospital-based cross-sectional study examined the prevalence of complicated appendicitis and the preoperative characteristics associated with it among children undergoing appendectomy. The cross-sectional label reflects the temporal structure of the primary question: complicated appendicitis is not incident during a period of observation but is already established when the child reaches the hospital, and surgery serves as the instrument that reveals it rather than as a follow-up endpoint. Presenting characteristics and disease severity were therefore ascertained within the same admission episode, and the analysis estimates prevalence and its correlates rather than incidence. The study was conducted at King Fahd Central Hospital in Jazan, Saudi Arabia, the principal tertiary referral centre for a population served by coastal, plain, and mountainous districts. Reporting follows the STROBE recommendations for observational studies [15]. Although the original protocol covered several hospitals within the Jazan Health Cluster, the present analysis draws exclusively on records from King Fahd Central Hospital.

### 2.2. Study Population and Eligibility Criteria

All children who underwent appendectomy between 1 January 2020 and 31 December 2024 and met the eligibility criteria were included. Children were eligible if they were aged 14 years or younger, underwent surgical treatment for acute appendicitis, and had complete medical records including operative documentation. Exclusions were age above 14 years, incidental appendectomy performed during another procedure, and substantial missing data on key variables or operative findings. A total of 384 paediatric appendectomy cases were identified during the study period and screened against the eligibility criteria by medical record review. Records held at Jazan General Hospital were also searched, but no file there retained the minimum set of variables required for the primary analysis, so the analytic cohort derives entirely from King Fahd Central Hospital. Following record review, 109 cases were excluded because of incomplete or insufficient medical record data, leaving 275 children in the final analysis. No formal sample-size calculation was performed because the study enumerated all eligible cases in the five-year period; with 54 events in the complete-case primary model, that model was constrained to nine covariates to maintain an acceptable events-per-variable ratio.

### 2.3. Definition of Disease Severity

Severity was classified from intraoperative findings. Complicated appendicitis was defined as documented appendiceal perforation, intra-abdominal abscess formation, or generalized peritonitis, consistent with the definitions used in contemporary paediatric series [3]. The primary outcome was defined strictly, and only on findings entered in the structured intraoperative record: a case was classified as complicated when appendiceal perforation, a purulent intra-abdominal collection, or generalized peritonitis was documented, and as uncomplicated otherwise. This definition was adopted so that every case could be classified by a single auditable rule applied uniformly to all records. Two broader definitions used in an earlier analysis of this cohort are retained as sensitivity analyses (Section 2.6). The first reproduces the original chart review, in which the operating team’s summary judgement was accepted and, where the operative description was ambiguous, placement of an intraoperative drain was treated as a surrogate for significant peritoneal contamination; 15 children were classified as complicated on that basis alone, and in a further 10, no perforation, purulent collection, generalized peritonitis, or drain placement was recorded in the structured fields. The second excludes those 15 drain-based classifications. Relative to the original chart review, the strict definition reclassifies 19 children as complicated in whom perforation, pus, or peritonitis was recorded in the structured fields but who had been summarised as uncomplicated, and reclassifies 25 as uncomplicated in whom no such finding was recorded.

### 2.4. Variables and Data Collection

Data were extracted from the hospital electronic medical record using a standardized abstraction protocol. Variables were grouped into the domains described below.

#### 2.4.1. Demographic and Admission Variables

Age, sex, nationality, weight, height, and body mass index were recorded, together with residence classified as urban or rural, mode of admission (referral from another facility or direct emergency department presentation), referral source (plain, coastal, or mountainous region, or direct admission), and time of admission (daytime or night-time).

#### 2.4.2. Symptom and Examination Variables

Duration of symptoms before admission was recorded in days. Presenting symptoms captured were abdominal pain, nausea, vomiting, anorexia, fever, diarrhoea, constipation, dysuria, and clinical dehydration as documented by the admitting clinician. Abdominal examination findings included right lower quadrant tenderness, guarding, rigidity, generalized abdominal tenderness, periumbilical tenderness, hypogastric tenderness, and the Blumberg, Rovsing, psoas, obturator, Dunphy, and McBurney signs. Admission vital signs comprised temperature, respiratory rate, peripheral oxygen saturation, pulse rate, and systolic and diastolic blood pressure, together with documented haemodynamic status.

#### 2.4.3. Laboratory and Imaging Variables

Complete blood count values at admission were extracted, including total leukocyte count, haemoglobin, mean corpuscular volume, and absolute neutrophil, lymphocyte, monocyte, eosinophil, and basophil counts. C-reactive protein, erythrocyte sedimentation rate, and lactate were recorded in fewer than one in five patients and were therefore excluded from analysis rather than imputed. Imaging variables included whether ultrasonography or computed tomography was performed, maximal appendiceal diameter in millimetres, and reported findings of perforation, abscess, appendicular mass, appendicolith, wall thickening, fat stranding, fluid collection, free fluid, mesenteric lymphadenopathy, and local inflammatory change. Maximal appendiceal diameter was recorded as the largest value reported by either modality and was not analysed separately by modality; of the 214 children with a recorded diameter, 127 had ultrasonography only, 41 computed tomography only, 36 had both, and in 10, the source modality was not recorded.

#### 2.4.4. Operative and Outcome Variables

Operative variables comprised the interval from admission to surgery in hours, operative approach (laparoscopic or open), timing of surgery, surgeon grade, appendix location, and drain placement. These were recorded to describe the management of the cohort and, in the case of the admission-to-surgery interval, tested as a potential correlate of disease severity.

### 2.5. Data Management and Quality Control

All identifiable information was removed before analysis and the dataset was stored on a password-protected system accessible only to the research team. Cleaning procedures identified and corrected inconsistencies and probable entry errors. Physiological outliers were reviewed against original clinical documentation and nursing records. Case-wise deletion was applied for multivariable analyses when values were missing on model variables. Recorded height was heaped at multiples of 10 cm in 223 of 275 children (81.1%), indicating estimation rather than measurement. Body mass index was therefore treated as unreliable: values outside a plausible paediatric range of 12 to 30 kg/m^2^ were flagged in 50 children (18.2%), body mass index is reported descriptively for the remaining 225 children only, and it was not entered into any model.

### 2.6. Statistical Analysis

Analyses were performed in R (version 4.2.3; R Foundation for Statistical Computing, Vienna, Austria). Continuous variables are presented as means with standard deviations and categorical variables as frequencies with percentages. The prevalence of complicated appendicitis is reported overall and by subgroup with Wilson score 95% confidence intervals, which perform better than the normal approximation at these sample sizes. Comparisons between children with uncomplicated and complicated appendicitis used the chi-square or Fisher’s exact test for categorical variables and independent-samples *t*-tests with Welch’s correction for continuous variables.

Because complicated appendicitis was common in this population, occurring in 20.7% of children, the odds ratio would materially overstate the underlying prevalence ratio. Effect estimates are therefore reported as adjusted prevalence ratios obtained from modified Poisson regression with a robust (sandwich) error variance, which estimates the ratio measure directly and remains reliable at the sample sizes available here [16]. Logistic regression models with identical specifications were fitted alongside as a sensitivity check; they identified the same variables with the same directions and are referred to where relevant.

Two multivariable models addressed the primary question. The first was restricted to preoperative variables and included age, sex, symptom duration, fever, clinical dehydration, generalized abdominal tenderness, guarding, leukocyte count, and haemoglobin. Covariates were chosen for clinical, biological, or logical plausibility rather than by bivariate significance alone, so that variables of established mechanistic relevance were retained irrespective of their unadjusted *p*-value. The three-day threshold for symptom duration was not prespecified in the study protocol. Symptom duration was therefore modelled continuously, with a quadratic term tested for nonlinearity, and the dichotomous form at three days, a cutoff in common clinical use, is retained as a secondary and clinically interpretable analysis; both are reported. Imaging findings that describe perforation, abscess, or appendicular mass were deliberately excluded from the multivariable models, since these radiological features describe the same pathological state used to define the outcome and their inclusion would be circular. A second model added maximal appendiceal diameter, restricted to children with a recorded measurement. Because this analysis rests on 37 events and selection into imaging was not random, it is reported as exploratory; the same model was refitted in the same children without diameter, so that the effect of the reduced sample could be separated from that of the added covariate. Two further sensitivity analyses repeated the primary model under the broader outcome definitions described in Section 2.3.

Adjusted prevalence ratios are reported with 95% confidence intervals. Multicollinearity was assessed using the variance inflation factor, with all values below 5 in both models. Explanatory power of the corresponding logistic specifications was summarized using Tjur’s coefficient of discrimination. All tests were two-tailed and a *p*-value below 0.05 was considered statistically significant.

### 2.7. Ethical Considerations

Ethical approval was obtained from the Research Ethics Committee of the Jazan Health Cluster, Saudi Arabia (approval number 2463, approved on 8 October 2024; National Committee of Bioethics registration number H-10-Z-141). The study was conducted in accordance with the Declaration of Helsinki. The committee waived the requirement for informed consent because the study was limited to the retrospective extraction of clinical data from existing patient files, with no access to personal identifying information and no contact with participants. Confidentiality was maintained throughout.

### 2.8. Use of Generative Artificial Intelligence

Generative artificial intelligence was used solely for language editing. The study design, data collection, statistical analysis, and interpretation were conducted entirely by the authors.

## 3. Results

### 3.1. Demographic and Admission Characteristics

A total of 275 children who underwent appendectomy were included. The mean age was 8.7 ± 2.4 years (range 3 to 14), and males predominated (n = 186, 67.6%). Most patients were Saudi nationals (n = 231, 84.0%), with 37 non-Saudi children (13.5%) and seven (2.5%) of unknown nationality or undocumented status. Mean weight was 27.6 ± 12.3 kg, mean height 121.9 ± 20.2 cm, and mean body mass index 18.9 ± 7.8 kg/m^2^. Height was recorded to the nearest 10 cm in most files, so body mass index should be read with caution; among the 225 children with a plausible value, mean body mass index was 17.9 ± 4.6 kg/m^2^ and did not differ by appendicitis type (18.1 ± 4.7 vs. 17.1 ± 4.2 kg/m^2^, *p* = 0.148).

Two thirds of the cohort arrived as referrals from peripheral facilities (n = 186, 67.6%), while 89 children (32.4%) presented directly to the emergency department. Among the 186 referrals, most originated from the plain region (n = 107, 57.5%), followed by the mountainous (n = 40, 21.5%) and coastal (n = 39, 21.0%) regions. A further six children who presented directly to the emergency department had a documented prior assessment at an outside facility; they are counted as direct presentations throughout, which reconciles the referral counts with the mode of admission. Residence was evenly split between rural (n = 138, 50.2%) and urban (n = 137, 49.8%) areas, and night-time admissions were more frequent than daytime admissions (58.2% vs. 41.8%) (Table 1).

### 3.2. Clinical Presentation

Median symptom duration before admission was 1 day (mean 2.0 ± 1.7 days), and 65 children (23.6%) presented three or more days after symptom onset. Abdominal pain was nearly universal (n = 269, 97.8%), followed by vomiting (n = 202, 73.5%), fever (n = 100, 36.4%), nausea (n = 96, 34.9%), and anorexia (n = 91, 33.1%). Diarrhoea (11.6%), dysuria (6.9%), constipation (5.1%), and clinical dehydration (3.6%) were uncommon.

**Figure 1:**
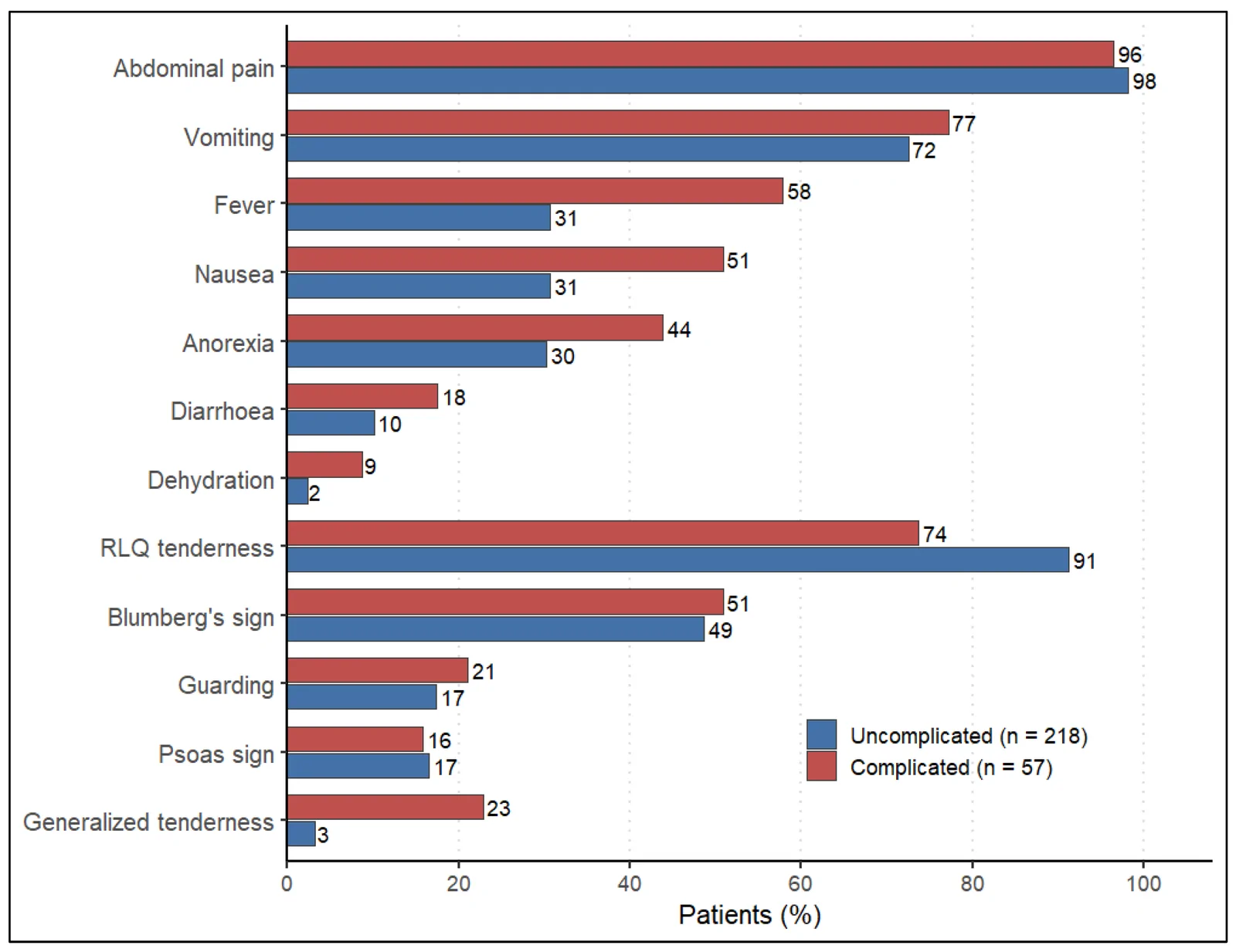
Presenting symptoms and abdominal examination findings by appendicitis type (N = 275).

On examination, right lower quadrant tenderness was documented in 241 children (87.6%) and rebound tenderness in 135 (49.1%). Guarding was present in 50 patients (18.2%), a positive psoas sign in 45 (16.4%), and generalized abdominal tenderness in 20 (7.3%). Almost all children were haemodynamically stable at admission (n = 269, 97.8%). Mean temperature was 36.9 ± 1.4 °C, pulse rate 97.7 ± 19.1 beats/min, and systolic blood pressure 105.1 ± 10.5 mmHg (Table 2, Figure 1).

### 3.3. Laboratory, Imaging, and Operative Profile

Mean leukocyte count was 13.2 ± 5.3 × 10^9^/L, with 87 children (31.6%) at or above 15 × 10^9^/L. Mean neutrophil count was 9.9 ± 7.2 × 10^9^/L and mean haemoglobin 11.6 ± 1.2 g/dL, with a mean corpuscular volume of 75.5 ± 7.2 fL. C-reactive protein and erythrocyte sedimentation rate were recorded in fewer than one in five patients and were therefore not analysed.

Ultrasonography was performed in 203 children (73.8%) and computed tomography in 93 (33.8%). Mean appendiceal diameter on imaging was 9.5 ± 4.1 mm (n = 214). Radiological perforation was reported in 19 patients (6.9%), abscess in 15 (5.5%), and appendicular mass in 11 (4.0%). Periappendiceal fluid collection was the most frequent imaging abnormality (n = 75, 27.3%).

Laparoscopic appendectomy was performed in 146 children (53.1%) and open appendectomy in 129 (46.9%). Mean interval from admission to surgery was 10.6 ± 8.0 h and mean operative time 70.2 ± 31.5 min. An intraoperative drain was placed in 35 patients (12.7%). Median hospital stay was 72 h (interquartile range 48 to 106) (Table 3).

### 3.4. Prevalence of Complicated Appendicitis

Structured intraoperative findings classified 57 of the 275 children as having complicated appendicitis. The prevalence of complicated appendicitis was therefore 20.7% (95% CI: 16.4–25.9). Radiological perforation had been reported preoperatively in 19 children, abscess in 15, and appendicular mass in 11, and an intraoperative drain was placed in 35. Under the two broader definitions retained as sensitivity analyses, prevalence was 22.9% (18.3–28.2) using the original chart review and 18.5% (14.2–23.6) when the 15 drain-based classifications were excluded.

Prevalence was stable across most demographic strata. It was 33.3% (95% CI: 18.6–52.2) in children aged five years and under, 20.8% (15.0–28.2) in those aged 6 to 9, and 17.3% (11.2–25.7) in those aged 10 to 14, with no significant difference across groups (*p* = 0.187). Prevalence was similar in girls and boys (19.1% vs. 21.5%, *p* = 0.763) and in referred and directly presenting children (21.5% vs. 19.1%, *p* = 0.763), and the difference by time from symptom onset did not reach significance (29.2% among children presenting three or more days after onset against 18.1% among those presenting earlier, *p* = 0.078). The one stratum across which prevalence differed significantly was residence, being higher among urban than rural residents (26.3%, 95% CI: 19.6–34.2, versus 15.2%, 10.2–22.1; *p* = 0.035) (Table 4).

### 3.5. Bivariate Associations with Complicated Appendicitis

Children with complicated appendicitis were slightly younger than those with uncomplicated disease (8.1 ± 2.4 vs. 8.9 ± 2.4 years, *p* = 0.038) and less often rural residents (36.8% vs. 53.7%, *p* = 0.035). The groups did not differ in sex distribution (70.2% vs. 67.0% male, *p* = 0.763), body mass index (*p* = 0.148), nationality (*p* = 0.334), or referral status (*p* = 0.763).

Differences emerged across the symptom, examination, and laboratory domains. Generalized abdominal tenderness showed the strongest categorical association (22.8% vs. 3.2%, χ^2^ = 22.91, *p* < 0.001), followed by reported fever (57.9% vs. 30.7%, χ^2^ = 13.26, *p* < 0.001) and nausea (50.9% vs. 30.7%, χ^2^ = 7.21, *p* = 0.007). Right lower quadrant tenderness ran in the opposite direction, being less frequent in complicated disease (73.7% vs. 91.3%, χ^2^ = 11.34, *p* < 0.001), while the Blumberg sign, guarding, and abdominal rigidity did not discriminate between groups. Presentation three or more days after symptom onset (33.3% vs. 21.1%, *p* = 0.078) and clinical dehydration (8.8% vs. 2.3%, *p* = 0.054) both approached but did not reach significance.

Admission temperature (37.2 ± 0.8 vs. 36.9 ± 1.6 °C, *p* = 0.013) and leukocyte count (15.0 ± 6.2 vs. 12.7 ± 4.9 × 10^9^/L, *p* = 0.014) were higher in complicated disease, whereas haemoglobin did not differ between groups (11.5 ± 1.2 vs. 11.6 ± 1.2 g/dL, *p* = 0.651). Appendiceal diameter was larger among complicated cases (11.3 ± 6.7 vs. 9.0 ± 3.0 mm, *p* = 0.040), and both appendicolith (24.6% vs. 6.4%, *p* < 0.001) and periappendiceal fluid collection (38.6% vs. 24.3%, *p* = 0.047) were more common. Radiological perforation, abscess, and appendicular mass were all associated with the intraoperative classification, as expected given that these findings describe the same pathological process used to define the outcome.

Pulse rate, respiratory rate, and oxygen saturation at admission did not differ between groups. The interval from admission to surgery was similar in both groups (10.1 ± 9.0 vs. 10.7 ± 7.8 h, *p* = 0.636), and operative approach was unrelated to severity (*p* = 0.334).

Length of hospital stay, recorded here as a descriptor of the clinical course rather than as an outcome under study, was longer among children with complicated appendicitis (115.9 ± 93.6 vs. 80.9 ± 57.3 h, *p* = 0.009) (Table 5).

### 3.6. Preoperative Characteristics Independently Associated with Complicated Appendicitis

The multivariable model included preoperative variables selected for clinical and biological plausibility rather than statistical significance alone: age, sex, symptom duration, fever, clinical dehydration, generalized abdominal tenderness, guarding, leukocyte count, and haemoglobin. Because complicated appendicitis occurred in 20.7% of children, adjusted prevalence ratios were estimated by modified Poisson regression with robust variance. The model included 267 patients with 54 events, and no multicollinearity was detected (maximum variance inflation factor 1.22). A logistic specification with identical covariates identified the same associations in the same directions (Tjur’s R^2^ = 0.174).

Two variables were independently associated with complicated appendicitis. Generalized abdominal tenderness tripled its prevalence (aPR = 3.01, 95% CI: 1.75–5.20, *p* < 0.001), and reported fever raised it by 87% (aPR = 1.87, 95% CI: 1.11–3.17, *p* = 0.019).

**Figure 2:**
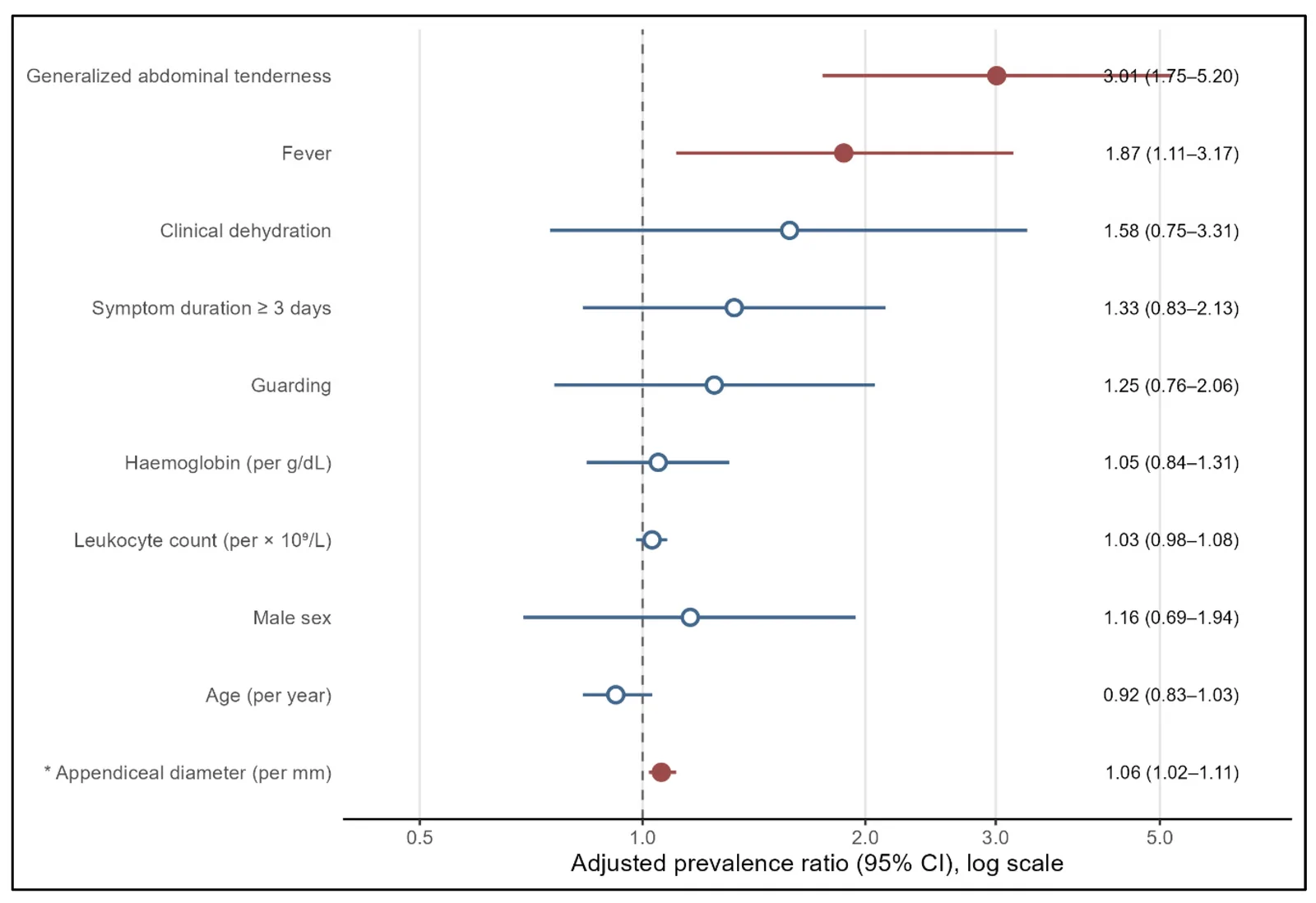
Adjusted prevalence ratios for preoperative characteristics independently associated with complicated appendicitis (red: significant; blue: non-significant). * Estimate from the exploratory model in Table 7 (n = 208); all other estimates from the primary model in Table 6 (n = 267).

Age, sex, symptom duration, clinical dehydration, guarding, leukocyte count, and haemoglobin were not independently associated with disease severity after adjustment. Symptom duration modelled continuously was likewise unassociated (aPR = 1.04 per day, 95% CI: 0.91–1.19, *p* = 0.600), and a quadratic term was not significant (*p* = 0.436); the three-day threshold therefore requires confirmation in an independent sample. Age and leukocyte count lost significance in the adjusted model despite significant bivariate differences (Table 6, Figure 2).

**Table 6:** Adjusted prevalence ratios for preoperative characteristics associated with complicated appendicitis (n = 267).

Variable	aPR	95% CI	*p*-Value
Age (per year)	0.92	0.83–1.03	0.139
Male sex (reference: female)	1.16	0.69–1.94	0.575
Symptom duration ≥ 3 days	1.33	0.83–2.13	0.242
Fever	1.87	1.11–3.17	0.019 *
Clinical dehydration	1.58	0.75–3.31	0.230
Generalized abdominal tenderness	3.01	1.75–5.20	<0.001 ***
Guarding	1.25	0.76–2.06	0.379
Leukocyte count (per × 10^9^/L)	1.03	0.98–1.08	0.236
Haemoglobin (per g/dL)	1.05	0.84–1.31	0.676

aPR, adjusted prevalence ratio from modified Poisson regression with robust variance; CI, confidence interval. * *p* < 0.05; ** *p* < 0.01; and *** *p* < 0.001. Maximum variance inflation factor 1.22. The corresponding logistic model identified the same associations (Tjur’s R^2^ = 0.174).

**Table 7:** Exploratory model including appendiceal diameter (n = 208 of the 214 children with a documented diameter).

Variable	aPR	95% CI	*p*-Value
Age (per year)	0.90	0.79–1.03	0.143
Male sex (reference: female)	0.98	0.51–1.88	0.943
Symptom duration ≥ 3 days	1.17	0.63–2.16	0.628
Fever	1.97	1.05–3.69	0.034 *
Clinical dehydration	0.91	0.30–2.69	0.860
Generalized abdominal tenderness	2.86	1.47–5.56	0.002 **
Leukocyte count (per × 10^9^/L)	1.03	0.97–1.10	0.283
Haemoglobin (per g/dL)	1.20	0.89–1.60	0.231
Appendiceal diameter (per mm)	1.06	1.02–1.11	0.003 **

aPR, adjusted prevalence ratio; CI, confidence interval. * *p* < 0.05; ** p<0.01, *** *p* < 0.001. Corresponding logistic model Tjur’s R^2^ = 0.187.

### 3.7. Exploratory Analysis Including Imaging

A second model added appendiceal diameter to the same clinical and laboratory covariates, restricted to children with a recorded measurement. Of the 214 children with a documented diameter, 208 had complete data on every model covariate and contributed to this analysis (37 events). Each additional millimetre of appendiceal diameter increased the prevalence of complicated appendicitis by 6% (aPR = 1.06, 95% CI: 1.02–1.11, *p* = 0.003). Generalized abdominal tenderness (aPR = 2.86, 95% CI: 1.47–5.56, *p* = 0.002) and reported fever (aPR = 1.97, 95% CI: 1.05–3.69, *p* = 0.034) both retained their associations (Table 7). This model is exploratory, resting on 37 events across nine covariates in a subgroup selected by the decision to image. Refitting it in the same 208 children without appendiceal diameter left both associations essentially unchanged (generalized tenderness aPR = 2.76, 1.45–5.23; fever aPR = 1.96, 1.03–3.75), indicating that diameter adds information without displacing the bedside signs. Children with and without a recorded diameter did not differ materially in age, symptom duration, leukocyte count, haemoglobin, sex, or the prevalence of complicated appendicitis (all *p* > 0.05).

### 3.8. Sensitivity Analyses of the Outcome Definition

The primary model was repeated under the two broader outcome definitions. Using the original chart review, in which the operating team’s summary judgement was accepted and drain placement served as a surrogate where documentation was ambiguous (n = 267, 60 events), symptom duration of three or more days (aPR = 1.70, 95% CI: 1.11–2.61, *p* = 0.016), clinical dehydration (aPR = 2.56, 1.32–4.99, *p* = 0.006), and generalized abdominal tenderness (aPR = 2.00, 1.13–3.54, *p* = 0.017) were independently associated with complicated disease, and each 1 g/dL of admission haemoglobin was associated with a 22% lower prevalence (aPR = 0.78, 0.66–0.93, *p* = 0.005). Excluding the 15 children classified on drain placement alone (n = 253, 46 events), symptom duration (aPR = 1.99, 1.20–3.30) and dehydration (aPR = 2.85, 1.43–5.68) strengthened, generalized tenderness weakened to borderline (aPR = 1.82, 0.95–3.49, *p* = 0.069), and the haemoglobin association was lost (aPR = 0.85, 0.67–1.07, *p* = 0.162) (Table 8). Generalized abdominal tenderness showed the most consistent association across the three outcome definitions, reaching significance under the primary and original-chart-review definitions and approaching it when drain-only classifications were excluded (aPR = 1.82, 0.95–3.49, *p* = 0.069); the symptom duration, dehydration, and haemoglobin associations were present only under the broader definitions.

### 3.9. Summary of Key Findings

The prevalence of complicated appendicitis was 20.7%. It did not vary by age, sex, or referral pathway, but was higher among urban residents. Two preoperative characteristics available at the bedside, generalized abdominal tenderness and reported fever, were independently associated with advanced disease, and appendiceal diameter added independent information where imaging had been performed (Figure 2). Generalized abdominal tenderness showed the most consistent association across the three outcome definitions examined.

## 4. Discussion

Among 275 children undergoing appendectomy in Jazan, complicated appendicitis was present in 20.7%. Generalized abdominal tenderness and reported fever were independently associated with advanced disease, and appendiceal diameter added information where imaging had been performed. Symptom duration, dehydration, haemoglobin, referral pathway, and the admission-to-surgery interval contributed nothing once these were accounted for, although the first three were associated with severity under broader outcome definitions.

### 4.1. Prevalence of Complicated Appendicitis in Context

The observed prevalence of 20.7% sits within the range reported for paediatric appendicitis, where roughly 20 to 35% of children have complicated disease at operation [2,3]. It is lower than the 30.9% reported from another Saudi centre [14], although the intervals overlap and case mix and referral thresholds are a likelier explanation than a regional contrast. Prevalence did not differ by age group, sex, or referral status, but was higher among urban than rural residents (26.3% versus 15.2%). That direction is opposite to what a distance-to-care account would predict and did not survive adjustment, so it is reported for completeness rather than as a geographic gradient.

### 4.2. Delayed Presentation and the Outcome Definition

Children presenting three or more days after symptom onset were more often classified as having complicated disease, but the difference was not significant and did not survive adjustment, and the confidence interval remains wide enough to accommodate a meaningful effect (aPR = 1.33, 95% CI: 0.83–2.13). Modelled continuously, each additional day carried no association at all. This is a null result against substantial literature: prospective data show perforation rising from 10% at 18 h to 44% by 36 h, with prehospital delay explaining most of the variance [9], and Pogorelić and colleagues found symptoms exceeding 24 h in 97% of complicated cases against 60% of uncomplicated ones [10]. Two features of these data may explain the discrepancy: duration was abstracted in whole days rather than hours, which attenuates any true gradient, and the association is sensitive to how severity is defined, reaching significance under both broader definitions (Table 8). Delayed presentation is therefore plausibly but not securely associated with severity here.

### 4.3. Generalized Tenderness, Fever, and Dehydration

Generalized abdominal tenderness tripled the prevalence of complicated disease and showed the most consistent association across the three outcome definitions examined, holding in the imaging model, though it did not reach significance when drain-only classifications were excluded. It is the physical sign of contamination beyond the right iliac fossa, and it contrasts instructively with the localizing signs: right lower quadrant tenderness was actually less frequent in complicated disease (73.7% versus 91.3%) and the Blumberg sign showed no association. The signs that establish the diagnosis of appendicitis are not the signs that grade it. Reported fever was the second independent correlate, consistent with a systemic response to established contamination, and measured temperature differed in the same direction. Both are obtainable without laboratory or imaging support. Clinical dehydration was more common in complicated disease but fell short of significance and did not survive adjustment. The rationale remains coherent, since protracted vomiting and third-space fluid loss accompany advanced disease and the same physiology underlies the hyponatremia-perforation association [13], but the estimate rests on ten affected children and reached significance only under the broader definitions, so it is too fragile to support a claim.

### 4.4. Haemoglobin, Laboratory Markers, and the Regional Anaemia Context

Admission haemoglobin was not associated with complicated appendicitis under the primary definition, in bivariate comparison or after adjustment. An inverse association was present under the broader definitions and disappeared once severity was restricted to objectively documented findings (Table 8), the clearest illustration in these data of how much the outcome definition matters. Haemoglobin is not an established marker of perforation and this analysis provides no support for treating it as one. The precedent is limited: Long and colleagues found lower haemoglobin, albumin, and prealbumin in children under four with perforated appendicitis, reading the pattern as a marker of nutritional reserve rather than of the appendicitis itself [12]. Mean haemoglobin was 11.6 g/dL with a mean corpuscular volume of 75.5 fL, consistent with the burden of haemoglobinopathy and iron deficiency in Jazan [17], so any signal is as likely to index chronic haematological disadvantage as anything about the appendix. Leukocyte count behaved similarly, differing between groups in bivariate comparison but carrying no independent association once fever and tenderness were in the model. Conventional parameters are a limited basis for grading severity, and the field has moved toward inflammatory biomarkers not measured routinely here. Interleukin-6 rises early and adds discriminatory information to standard blood tests [18], and paediatric data show higher levels in complicated disease, although its capacity alone is moderate and it is not an established independent predictor [19]. Neither it nor C-reactive protein was available here; combining them prospectively with the bedside signs identified above is the natural next step.

### 4.5. Appendiceal Diameter

In the 208 children who contributed to this model, each additional millimetre of appendiceal diameter was associated with a 6% higher prevalence of complicated appendicitis. The direction matches the CLU score derivation, in which a threshold of 8.45 mm was among ten features distinguishing complicated disease in children [11]; mean diameter here was 11.3 mm in complicated cases against 9.0 mm in uncomplicated ones, straddling that threshold. The practical value is qualified by coverage, since diameter was documented in 78% of children and ultrasonography performed in 74%. Because ultrasound and computed tomography measurements were pooled and the model is exploratory, the estimate indicates a direction rather than a calibrated per-millimetre risk.

### 4.6. Characteristics Not Associated with Severity

Referral source, mode of admission, and nationality were unrelated to severity, and the unadjusted difference by residence did not persist after adjustment. Disparities in the emergency care of children are documented elsewhere [20], so their absence here is worth stating rather than assuming. Vital signs showed no association, consistent with 97.8% of the sample being stable on arrival. The admission-to-surgery interval was unrelated to severity, which accords with evidence that short, structured in-hospital delays do not worsen outcomes when resuscitation and antibiotics begin on arrival [7,8]. That comparison should be read cautiously: the classification is made at the end of the interval, so the absence of an association does not establish that shorter delays cannot affect progression.

### 4.7. Clinical Implications

The characteristics identified here require neither imaging nor laboratory turnaround, which suits them to first contact rather than the operating theatre. A child arriving with reported fever and tenderness beyond the right iliac fossa may warrant heightened suspicion of advanced disease, with prompt resuscitation, antibiotics, and surgical assessment. This study did not evaluate a triage strategy or show that differential prioritization improves outcomes, so these findings support clinical vigilance rather than a validated protocol. Severity stratification also bears on treatment selection, since nonoperative management with antibiotics is accepted for uncomplicated disease [21] while perforated appendicitis follows a different logic [22]. Because symptom duration and the admission-to-surgery interval were both unassociated under the primary definition, these data do not identify either as the more promising target.

### 4.8. Strengths and Limitations

The study enumerated every eligible paediatric appendectomy over five consecutive years rather than sampling. Severity was classified from structured intraoperative findings by a single explicit rule rather than from imaging or discharge coding, explanatory variables were restricted to the preoperative period, and radiological correlates of the outcome were excluded to guard against circularity. Reporting the same models under three definitions makes the dependence of each association on that definition visible.

The cross-sectional design is the most important limitation. Because presenting characteristics and severity were ascertained within the same admission episode, the analysis establishes association rather than temporal sequence. Generalized tenderness is the clearest example: it is a sign of established contamination rather than a characteristic preceding it, so its association carries no implication that acting earlier would prevent progression. The estimate describes children who came to appendectomy rather than all children with appendicitis in the region, and the single-centre retrospective design limits generalizability. Outcome classification is the second: the associations with symptom duration, dehydration, and haemoglobin were significant under the broader definitions and absent under the primary one (Table 8), and while the strict definition is more defensible, it depends on the completeness of the structured operative record. C-reactive protein, erythrocyte sedimentation rate, and serum sodium were unavailable, which probably explains part of the modest explanatory power (Tjur’s R^2^ = 0.174). The three-day cutoff was not prespecified, and height was recorded to the nearest 10 cm in most files, so body mass index is unreliable and was not modelled. The period spans the COVID-19 pandemic, during which delayed presentations and perforation rates rose in many health systems; date of admission was not collected, so a temporal analysis could not be performed.

A prospective registry capturing hourly symptom duration, serum sodium, and C-reactive protein, with the outcome classified at operation, is the only way to settle whether delayed presentation and dehydration carry the associations the broader definitions suggest.

## 5. Conclusions

One in five children undergoing appendectomy at this tertiary referral centre had complicated appendicitis, a prevalence of 20.7% that was uniform across age, sex, and referral pathway. Two characteristics obtainable at the bedside were independently associated with advanced disease: generalized abdominal tenderness and reported fever. Appendiceal diameter contributed further where imaging had been performed. Associations with delayed presentation, clinical dehydration, and admission haemoglobin appeared under broader definitions of complicated disease and did not persist when severity was restricted to objectively documented operative findings, so they should be treated as hypothesis-generating. Neither the interval from admission to surgery nor the operative approach was associated with severity, although this design cannot establish that in-hospital waiting has no effect on progression. Recognizing advanced disease at first contact is the priority these data support. Shortening the interval from symptom onset to specialist assessment remains reasonable on the basis of the wider paediatric literature, but symptom duration was not associated with severity in this cohort, and that recommendation does not follow from the present findings. The model stratifies risk rather than diagnosing severity, and should be read as a guide to clinical suspicion rather than as a substitute for operative assessment.

## Figures and Tables

**Table 1:** Demographic and admission characteristics of participants (N = 275).

Variable	Mean ± SD/n (%)
Age (years)	8.7 ± 2.4 (3–14)
Weight (kg)	27.6 ± 12.3 (10–85)
Height (cm)	121.9 ± 20.2 (50–164) †
Body mass index (kg/m^2^) †	17.9 ± 4.6 (12.0–29.9) †
**Sex**	
Male	186 (67.6)
Female	89 (32.4)
**Nationality**	
Saudi	231 (84.0)
Non-Saudi	37 (13.5)
Unknown or undocumented	7 (2.5)
**Mode of admission**	
Referred	186 (67.6)
Emergency department	89 (32.4)
**Referral source**	
Plain region (referred)	107 (38.9)
Direct admission	89 (32.4)
Coastal region (referred)	39 (14.2)
Mountainous region (referred)	40 (14.5)
**Residence**	
Rural	138 (50.2)
Urban	137 (49.8)
**Time of admission**	
Night	160 (58.2)
Day	115 (41.8)

SD, standard deviation. Ranges are shown in parentheses for continuous variables. † Height was recorded to the nearest 10 cm in 81.1% of files; body mass index is therefore shown for the 225 children with a plausible value of 12 to 30 kg/m^2^ and was not entered into any model.

**Table 2:** Clinical presentation and admission vital signs (N = 275).

Variable	Mean ± SD/n (%)
Symptom duration (days)	2.0 ± 1.7
Symptom duration ≥ 3 days	65 (23.6)
**Presenting symptoms**	
Abdominal pain	269 (97.8)
Vomiting	202 (73.5)
Fever	100 (36.4)
Nausea	96 (34.9)
Anorexia	91 (33.1)
Diarrhoea	32 (11.6)
Dysuria	19 (6.9)
Constipation	14 (5.1)
Clinical dehydration	10 (3.6)
**Abdominal examination findings**	
Right lower quadrant tenderness	241 (87.6)
Blumberg’s sign	135 (49.1)
Guarding	50 (18.2)
Psoas sign	45 (16.4)
Rovsing’s sign	36 (13.1)
Obturator sign	27 (9.8)
Generalized abdominal tenderness	20 (7.3)
McBurney’s sign	17 (6.2)
Dunphy’s sign	16 (5.8)
Abdominal rigidity	13 (4.7)
**Past medical history**	
Previous infection	13 (4.7)
Atopy	12 (4.4)
**Haemodynamic status**	
Stable	269 (97.8)
Unstable	6 (2.2)
**Admission vital signs**	
Temperature (°C)	36.9 ± 1.4
Respiratory rate (breaths/min)	21.2 ± 9.0
Peripheral oxygen saturation (%)	98.6 ± 8.1
Pulse rate (beats/min)	97.7 ± 19.1
Systolic blood pressure (mmHg)	105.1 ± 10.5
Diastolic blood pressure (mmHg)	63.3 ± 10.9

**Table 3:** Laboratory, imaging, and operative profile (N = 275).

Variable	Mean ± SD/n (%)
**Laboratory values at admission**	
Leukocyte count (×10^9^/L)	13.2 ± 5.3
Leukocyte count ≥ 15 × 10^9^/L	87 (31.6)
Neutrophil count (×10^9^/L)	9.9 ± 7.2
Lymphocyte count (×10^9^/L)	2.2 ± 1.1
Haemoglobin (g/dL)	11.6 ± 1.2
Mean corpuscular volume (fL)	75.5 ± 7.2
**Imaging**	
Ultrasonography performed	203 (73.8)
Computed tomography performed	93 (33.8)
Appendiceal diameter (mm), n = 214	9.5 ± 4.1
Fluid collection	75 (27.3)
Mesenteric lymph nodes	39 (14.2)
Free fluid	37 (13.5)
Fat stranding	37 (13.5)
Thickened wall	35 (12.7)
Appendicolith	28 (10.2)
Perforation	19 (6.9)
Abscess	15 (5.5)
Local inflammatory changes	13 (4.7)
Appendicular mass	11 (4.0)
**Operative details**	
Laparoscopic approach	146 (53.1)
Open approach	129 (46.9)
Night-time surgery	146 (53.1)
Consultant surgeon	178 (64.7)
Drain placed	35 (12.7)
Admission-to-surgery interval (hours)	10.6 ± 8.0
Operative duration (minutes)	70.2 ± 31.5
**Appendicitis type**	
Uncomplicated	218 (79.3)
Complicated	57 (20.7)

**Table 4:** Prevalence of complicated appendicitis by subgroup (N = 275).

Subgroup	n	Complicated, n (%)	95% CI	*p*-Value
**Overall**	**275**	**57 (20.7)**	**16.4–25.9**	**—**
**Age group**				
≤5 years	27	9 (33.3)	18.6–52.2	
6–9 years	144	30 (20.8)	15.0–28.2	
10–14 years	104	18 (17.3)	11.2–25.7	0.187
**Sex**				
Female	89	17 (19.1)	12.3–28.5	
Male	186	40 (21.5)	16.2–28.0	0.763
**Residence**				
Rural	138	21 (15.2)	10.2–22.1	
Urban	137	36 (26.3)	19.6–34.2	0.035 *
**Mode of admission**				
Referred	186	40 (21.5)	16.2–28.0	
Emergency department	89	17 (19.1)	12.3–28.5	0.763
**Symptom duration**				
<3 days	210	38 (18.1)	13.5–23.9	
≥3 days	65	19 (29.2)	19.6–41.2	0.078

CI, Wilson score confidence interval. *p*-values from the chi-square test across the categories of each subgroup * *p* < 0.05.

**Table 5:** Bivariate association between study variables and appendicitis type, with preoperative characteristics and operative or postoperative descriptors listed separately.

Variable	Uncomplicated (n = 218)	Complicated (n = 57)	Test	*p*-Value
Age (years)	8.9 ± 2.4	8.1 ± 2.4	t = 2.11	0.038 *
Male sex	146 (67.0)	40 (70.2)	χ^2^ = 0.09	0.763
Saudi nationality	186 (85.3)	45 (78.9)	χ^2^ = 0.93	0.334
Body mass index (kg/m^2^), n = 225	18.1 ± 4.7	17.1 ± 4.2	t = 1.46	0.148
Referred admission	146 (67.0)	40 (70.2)	χ^2^ = 0.09	0.763
Rural residence	117 (53.7)	21 (36.8)	χ^2^ = 4.47	0.035 *
Symptom duration (days)	1.9 ± 1.6	2.4 ± 2.1	t = −1.75	0.084
Symptom duration ≥ 3 days	46 (21.1)	19 (33.3)	χ^2^ = 3.10	0.078
Vomiting	158 (72.5)	44 (77.2)	χ^2^ = 0.30	0.583
Nausea	67 (30.7)	29 (50.9)	χ^2^ = 7.21	0.007 **
Anorexia	66 (30.3)	25 (43.9)	χ^2^ = 3.18	0.075
Fever	67 (30.7)	33 (57.9)	χ^2^ = 13.26	<0.001 ***
Diarrhoea	22 (10.1)	10 (17.5)	χ^2^ = 1.77	0.183
Dysuria	13 (6.0)	6 (10.5)	χ^2^ = 0.84	0.360
Clinical dehydration	5 (2.3)	5 (8.8)	χ^2^ = 3.72	0.054
Right lower quadrant tenderness	199 (91.3)	42 (73.7)	χ^2^ = 11.34	<0.001 ***
Blumberg’s sign	106 (48.6)	29 (50.9)	χ^2^ = 0.02	0.877
Guarding	38 (17.4)	12 (21.1)	χ^2^ = 0.19	0.661
Generalized abdominal tenderness	7 (3.2)	13 (22.8)	χ^2^ = 22.91	<0.001 ***
Abdominal rigidity	10 (4.6)	3 (5.3)	χ^2^ = 0.00	1.000
Temperature (°C)	36.9 ± 1.6	37.2 ± 0.8	t = −2.50	0.013 *
Pulse rate (beats/min)	97.3 ± 19.7	99.5 ± 16.4	t = −0.88	0.381
Respiratory rate (breaths/min)	21.5 ± 9.7	19.9 ± 5.2	t = 1.71	0.090
Oxygen saturation (%)	98.5 ± 9.1	99.1 ± 1.0	t = −0.92	0.358
Leukocyte count (×10^9^/L)	12.7 ± 4.9	15.0 ± 6.2	t = −2.53	0.014 *
Neutrophil count (×10^9^/L)	9.6 ± 7.5	11.1 ± 6.1	t = −1.57	0.120
Haemoglobin (g/dL)	11.6 ± 1.2	11.5 ± 1.2	t = 0.45	0.651
Appendiceal diameter (mm)	9.0 ± 3.0	11.3 ± 6.7	t = −2.12	0.040 *
Appendicolith	14 (6.4)	14 (24.6)	χ^2^ = 14.33	<0.001 ***
Fluid collection	53 (24.3)	22 (38.6)	χ^2^ = 3.96	0.047 *
Fat stranding	25 (11.5)	12 (21.1)	χ^2^ = 2.79	0.095
Perforation on imaging †	8 (3.7)	11 (19.3)	χ^2^ = 14.82	<0.001 ***
Abscess on imaging †	5 (2.3)	10 (17.5)	χ^2^ = 17.53	<0.001 ***
Appendicular mass †	5 (2.3)	6 (10.5)	χ^2^ = 5.98	0.015 *
**Operative and postoperative descriptors (not preoperative characteristics)**				
Open appendectomy	106 (48.6)	23 (40.4)	χ^2^ = 0.93	0.334
Admission-to-surgery interval (hours)	10.7 ± 7.8	10.1 ± 9.0	t = 0.48	0.636
Hospital stay (hours)	80.9 ± 57.3	115.9 ± 93.6	t = −2.70	0.009 **

Values are mean ± SD or n (%). * *p* < 0.05; ** *p* < 0.01; and *** *p* < 0.001. † Radiological features that describe the same pathological state used to define the outcome, not entered into the multivariable models. Percentages are column percentages calculated within each appendicitis group, so counts in the two columns sum to the totals reported in Table 1.

**Table 8:** Adjusted prevalence ratios under three definitions of complicated appendicitis (sensitivity analyses).

Variable	Primary (Strict) aPR (95% CI)	Original Chart Review aPR (95% CI)	Excluding Drain-Only aPR (95% CI)
Age (per year)	0.92 (0.83–1.03)	1.02 (0.94–1.11)	1.04 (0.94–1.15)
Male sex (reference: female)	1.16 (0.69–1.94)	0.98 (0.63–1.54)	0.92 (0.54–1.57)
Symptom duration ≥ 3 days	1.33 (0.83–2.13)	1.70 (1.11–2.61) *	1.99 (1.20–3.30) **
Fever	1.87 (1.11–3.17) *	1.31 (0.82–2.10)	1.46 (0.81–2.61)
Clinical dehydration	1.58 (0.75–3.31)	2.56 (1.32–4.99) **	2.85 (1.43–5.68) **
Generalized abdominal tenderness	3.01 (1.75–5.20) ***	2.00 (1.13–3.54) *	1.82 (0.95–3.49)
Guarding	1.25 (0.76–2.06)	1.47 (0.95–2.28)	1.51 (0.90–2.53)
Leukocyte count (per × 10^9^/L)	1.03 (0.98–1.08)	1.03 (0.99–1.07)	1.05 (1.00–1.10) *
Haemoglobin (per g/dL)	1.05 (0.84–1.31)	0.78 (0.66–0.93) **	0.85 (0.67–1.07)
Observations/events	267/54	267/60	253/46

aPR, adjusted prevalence ratio from modified Poisson regression with robust variance; CI, confidence interval. * *p* < 0.05; ** *p* < 0.01; and *** *p* < 0.001. The primary (strict) definition restricts complicated appendicitis to children with perforation, purulent intra-abdominal collection, or generalized peritonitis recorded in the structured operative fields. The original chart review accepted the operating team’s summary judgement and used drain placement as a surrogate where documentation was ambiguous. The third column excludes the 15 children classified on drain placement alone.

## Data Availability

The data presented in this study are available on request from the corresponding author due to the fact that they were extracted from paediatric medical records and contain potentially identifying clinical information.

## Abbreviations

The following abbreviations are used in this manuscript:

aPR	Adjusted prevalence ratio
BMI	Body mass index
CI	Confidence interval
CRP	C-reactive protein
CT	Computed tomography
ESR	Erythrocyte sedimentation rate
IQR	Interquartile range
MCV	Mean corpuscular volume
OR	Odds ratio
SD	Standard deviation
